# Cytotoxic Activity of Saponins and Sapogenins Isolated from *Chenopodium quinoa* Willd. in Cancer Cell Lines

**DOI:** 10.1155/2023/8846387

**Published:** 2023-12-18

**Authors:** Genesis N. Carpio-Paucar, Andrea I. Palo-Cardenas, Alejandro N. Rondón-Ortiz, Alejandro Pino-Figueroa, Elvis Gilmar Gonzales-Condori, José A. Villanueva-Salas

**Affiliations:** ^1^MCPHS University, 179 Longwood Avenue, Boston, MA 02115, USA; ^2^Universidad Tecnológica del Perú (UTP), Av. Tacna y Arica 160, Arequipa, Peru; ^3^Universidad Católica de Santa María, Urb. San Jose s/n, Arequipa, Peru

## Abstract

The cytotoxic properties of two extracts from *Chenopodium quinoa* Willd. and three synthetic sapogenins were evaluated in different cancer cell lines (A549, SH-SY5Y, HepG2, and HeLa) to investigate their cytotoxic effects and determine if these cell lines activate the caspase pathway for apoptosis in response to saponin and sapogenin treatment. The saponin extracts were isolated from the agro-industrial waste of *Chenopodium quinoa* Willd., while the sapogenins were identified and quantitatively determined by High-Performance Liquid Chromatography (HPLC). Among these compounds, ursolic acid was the most active compound, with high IC_50_ values measured in all cell lines. In addition, hederagenin demonstrated higher caspase-3 activity than staurosporine in HeLa cells, suggesting an anti-cytotoxic activity via a caspase-dependent apoptosis pathway. HPLC analysis showed that the concentration of hederagenin was higher than that of oleanolic acid in ethanolic extracts of white and red quinoa. The ethanolic extracts of white and red quinoa did not show cytotoxic activity. On the other hand, the synthetic sapogenins such as ursolic acid, oleanolic acid, and hederagenin significantly decreased the viability of the four cell lines studied. Finally, by Caspase-3 assay, it was found that HeLa undergoes apoptosis during cell death because hederagenin produces a significant increase in PARP-1 hydrolysis in HeLa cells.

## 1. Introduction

The World Health Organization has reported that cancer is one of the ten most frequent causes of death worldwide, with a drastic increase in its incidence. Given that conventional modalities for the treatment of cancer are invasive and expensive, new alternatives have been proposed and the interest in medicinal plants as possible sources of compounds with anticancer activity has increased. Quinoa grain (*Chenopodium quinoa* Willd.) is known for its high nutritional content which is a functional and ideal food for human beings. Quinoa grain contains 18% protein and is a rich source of fiber, antioxidants, and minerals; moreover, it is gluten-free and contains vitamins such as A, B2, C, E, thiamine, and folic acid. The fats present in quinoa include omega 6, omega 9, omega 3, and palmitic acid, and it contains between 58 and 68% carbohydrates [1–5]. However, quinoa contains several compounds that decrease its nutritional quality, one of which is the saponin located in the pericarp of the seeds. Saponins are abundant antinutrients that are intensely bitter compounds and potentially toxic if ingested in large quantities and thus function as protection against birds, insects, and fungi [6].

The varied composition of *Chenopodium quinoa* Willd. could provide this food with different properties such as an anticarcinogenic effect, since it has been demonstrated that the phenolic compounds of quinoa have an inhibitory effect on the proliferation of AT-2 and MAT-LyLu rat prostate cancer cells [7]. Another study demonstrated that quinoa proteins exhibit viability inhibitory properties in human colorectal cancer cell lines (Caco-2, HT-29, and HCT-116) [8]. Likewise, quinoa seed powder showed cytotoxicity against the hepatocarcinoma cell line HEPG2 and would provide hepatoprotection against non-alcoholic fatty liver disease [9]. Also, quinoa protein or its hydrolysate was shown to improve the azoxymethane/sodium dextran sulfate-induced colorectal cancer mouse model by altering the intestinal flora and increasing the production of beneficial short-chain fatty acids [10]. On the other hand, a new polysaccharide made up of galacturonic acid and glucose isolated from quinoa showed an anticancer effect in human liver cancer SMMC 7721 and breast cancer MCF-7 cells [11]. Black quinoa seed oil had an antiproliferative effect on HCT 116 (human colon carcinoma) cells by inducing apoptosis in a dose-dependent manner [12]. Finally, the aglycones of quinoa saponins that are triterpenoids called triterpenoid saponins would show anticancer and anti-inflammatory effects [13, 14].

Quinoa contains at least 30 different types of saponins derived from sapogenins such as hederagenin, oleanolic acid, serjanic acid, and phytolaccagenic acid [6, 15, 16]. Because these compounds possess broad properties, the objective of this research was to evaluate the cytotoxic effects of saponins and sapogenins isolated from *Chenopodium quinoa* Willd. on different cancer cell lines to provide a basis and additional information for future preclinical cancer research.

## 2. Materials and Methods

### 2.1. General Experimental Procedures

All solvents and reagents were purchased from commercial sources and used directly without further purification. The starting plant material was provided as a fine powder of two types of *Chenopodium quinoa* Willd. saponins (red and white quinoa), obtained by the company JIWRA SAC (Arequipa, Peru). High-Performance Liquid Chromatography (HPLC) was conducted on a Hitachi Primaide with a DAD detector Primaide 1430 and a Thermo Scientific™ Hypersil GOLD™ C18 Reversed Phase HPLC Column, 5 *μ*m, 2.1 mm × 20 mm. The purity of all compounds was >95% as determined by HPLC. Methanol : acid water (89 : 11) was used as a mobile phase with a flow rate of 1.5 mL/min. The Thin Layer Chromatography (TLC) analysis was carried out on silica gel plates that were visualized under UV at 366 nm and sprayed with Liebermann–Burchard reagent before heating.

### 2.2. Preparation of Plant Extracts

Extraction of the active compounds of *Chenopodium quinoa* Willd. was performed using a percolation method with 95% ethanol. Both plant samples were treated separately. The plant matrix was suspended in the solvent for 24 h, and then the extracts were drained at a flow rate of 60 drops/min. Finally, the ethanol was evaporated with a rotary evaporator system (BÜCHI).

### 2.3. Cell Culture

The A549 human lung carcinoma, HeLa human cervical cancer, SH-SY5Y neuroblastoma cancer, and HepG2 human hepatocellular carcinoma cell lines were obtained from the American Type Culture Collection (ATCC) and cultured as monolayers in Dulbecco's Modified Eagle's Medium (Sigma-Aldrich). The medium was supplemented with 10% fetal bovine serum (FBS) (ATCC) and 5 mL penicillin/streptomycin (Gibco) and incubated at 37°C, 95% relative humidity, and 5% CO_2_.

### 2.4. Cytotoxicity Measurements

The cytotoxicity of the two ethanolic extracts and the three synthetic sapogenins, ursolic acid (Selleckchem), oleanolic acid (Selleckchem), and hederagenin (Selleckchem), were measured in vitro using the 3-(4,5-dimethylthiazol-2-yl)-5-(3-carboxymethoxyphenyl)-2-(4-sulfophenyl)-2H-tetrazolium (MTS) assay (Promega) [17–21]. Both the extracts and the sapogenins were prepared by diluting them in 5% (v/v) of dimethyl sulfoxide (DMSO) (Sigma-Aldrich). Cells were seeded at a concentration of 10 000 cells per well in 96-well plates. After incubation overnight in fetal bovine serum low medium (1% FBS), the ethanolic extracts (0; 0.3; 1; 3; 10; 30; 100 mg/L) and the synthetic compounds (0; 1; 3; 10; 30; 100; 300 *µ*M) were added to the cells at various concentrations. After 24 h, the extracts and compounds were removed and the MTS reagent was added; the plates were incubated at 37°C, 5% CO_2_, and 95% relative humidity for another 3 h before the absorbance was measured at 490 nm using a plate reader spectrophotometer (Biotek Synergy HT) and the mean absorbance values were calculated. The cytotoxic activities were expressed as IC_50_, which represented the cell viability percentage after the treatments compared to the untreated controls. Dose-response curves were plotted for the samples. All assays were performed in triplicate.

### 2.5. Caspase-3 Activity

The caspase-3 activity in A549, HeLa, SH-SY5Y, and HepG2 cells was measured with the caspase-3 colorimetric assay kit (BioVision K-106) [22]. After treatment with the staurosporine [23] positive control and the three synthetic sapogenins at different concentrations, the four cell lines were harvested and lysed on ice. The protein in the cell lysates was quantified using the BCA (bicinchoninic acid) assay and adjusted to the suggested concentration before the caspase activity was measured. Caspase-3 recognizes the DEVD sequence and cleaves the provided DEVD-pNA substrate, which produces the p-nitroaniline chromophore that emits a quantifiable light signal that can be measured at 405 nm by a spectrophotometer. All assays were performed in triplicate.

### 2.6. Western Blotting Analysis

Half a million HeLa cells were seeded in 6-well culture dishes and were treated with different concentrations of ursolic acid (UA), oleanolic acid (OA), or hederagenin (HED) for 5 and 24 h. The medium was then removed and the cells were washed twice with 500 *μ*L of cold PBS (phosphate buffered saline) (4°C) before 300 *μ*L of MPER cell lysis reagent (Thermo Fisher Scientific) was added to each well and incubated for 5 min at 4°C. The wells were scraped to collect the cells, which were transferred to microcentrifuge tubes. Lysed proteins were isolated by centrifugation and quantified using a BCA assay. The same volume of proteins was separated by electrophoresis on a 7.5% polyacrylamide gel (Bio-Rad) and then subjected to electroblotting on a 0.45 *µ*m pore diameter nitrocellulose membrane. The blots were probed with anti-cleaved PARP-1 (poly(ADP-ribose) polymerase-1) (Abcam; 1 : 5000), anti-PARP-1 (Abcam; 1 : 5000), *β*-actin (Abcam; 1 : 10000), and goat polyclonal antibody to rabbit IgG (HRP) (Abcam; 1 : 5000). Reactive bands were detected by chemiluminescence on a C-DiGit blot scanner (LI-COR Biosciences). Images were captured, stored, and analyzed with the “Image Studio Digits ver. 5.0” program.

### 2.7. Obtaining Saponins via Liquid-Liquid Extraction

Five grams of the plant sample concentrate were weighed and dissolved in 15 mL of distilled water and poured into a decantation funnel where it was subjected to three continuous extractions with 10 mL of chloroform each. The aqueous phase was recovered and extracted three times with 10 mL of ethyl acetate each to eliminate the oily phase. Finally, three extractions with 1-butanol were done to eliminate the aqueous phase. The oily phase was collected and placed in an oven at 24–90°C until complete dryness. The powder obtained was then dissolved in 10 mL of 1% NaOH, and three more extractions were conducted with 5 mL of 1-butanol. The butanol fractions were put back into the oven set to the conditions indicated above to obtain the purified saponins. Both isolates from white and red quinoa were subjected to this procedure.

### 2.8. Percolation of Sapogenins by Acid Hydrolysis

One hundred milligrams of saponins isolated from red and white quinoa were weighed separately and dissolved in 25 mL of distilled water and heated in a water bath; 10 mL of concentrated hydrochloric acid was added to reflux for 20 min. The pH reaction was adjusted with 30% sodium hydroxide before the solutions were added to a decantation funnel in which three successive extractions were carried out with 10 mL of chloroform. The chloroform phase was collected and placed in an oven until the solvent was completely evaporated and a dry powder was obtained.

### 2.9. HPLC Analysis

Hederagenin and oleanolic acid stock solutions of 2, 4, 8, 16, and 32 mg/L were used to calibrate the system. Additionally, 600 *µ*L of saponins isolated from each of the quinoas was added to 2 mL of methanol and was filtered through a glass syringe into sample vials. Throughout the development of the experiment, ≥99% HPLC-grade methanol was used. Methanol : acid water (89 : 11) was used as a phase solution for HPLC experiments. It was prepared by diluting orthophosphoric acid in ultrapure water, to which 20 *µ*L of each synthetic sapogenin was loaded into a GOLD Hypersil-5 *µ*m column C-18 (Thermo Fisher Scientific), with a 10 min run at 20°C and 1.5 mL/min flow rate. Each run was performed in triplicate, and the wavelength for the detection of hederagenin and oleanolic acid was 210 nm.

## 3. Results

### 3.1. In Vitro Cytotoxic Activity of *Chenopodium quinoa* Willd. Ethanolic Extracts

The cytotoxic activity of the ethanolic extracts from red and white quinoa was evaluated in four different cancer cell lines using a concentration range of 0–100 mg/L in an MTS assay. The results demonstrated that neither of the two ethanolic extracts reduced cell viability in the cell lines tested (data not shown), indicating that both the white and red quinoa extracts had no cytotoxic effect, and therefore we did not test further.

### 3.2. The Effect of Ursolic Acid on the Cell Viability of A549, HeLa, SH-SY5Y, and HepG2 Cell Lines

**Figure 1:**
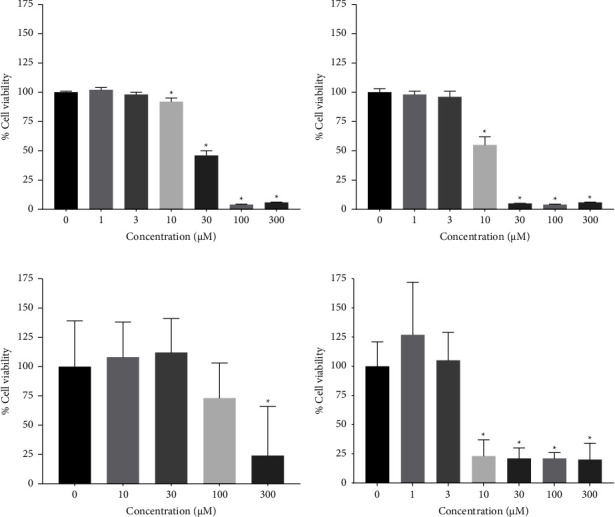
The cytotoxic effect of 0 *µ*M–300 *µ*M ursolic acid in cell lines A549 (a), HeLa (b), HepG2 (c), and SH-SY5Y (d) after 24 h.

The effect of ursolic acid (UA) on cell viability was evaluated using the MTS assay in four cancer cell lines. The results are presented as the concentration of UA that inhibited the growth of the cells by 50% (IC_50_). The cell viability was reduced in cancer cell lines treated with 0–300 *µ*M UA, suggesting that UA had a cytotoxic effect at concentrations of 30 *µ*M, 10 *µ*M, 300 *µ*M, and 10 *µ*M on A549, HeLa, HepG2, and SH-SY5Y cell lines, respectively (Figure 1). The IC_50_ values were 21.9 ± 0.05 *μ*M for A549, 11.2 ± 0.05 *μ*M for HeLa, and 104.2 ± 0.05 *μ*M for HepG2, with the highest activity of 6.9 ± 0.05 *µ*M in SH-SY5Y cells.

### 3.3. Effect of Oleanolic Acid on Cell Viability in Cancer Cell Lines

**Figure 2:**
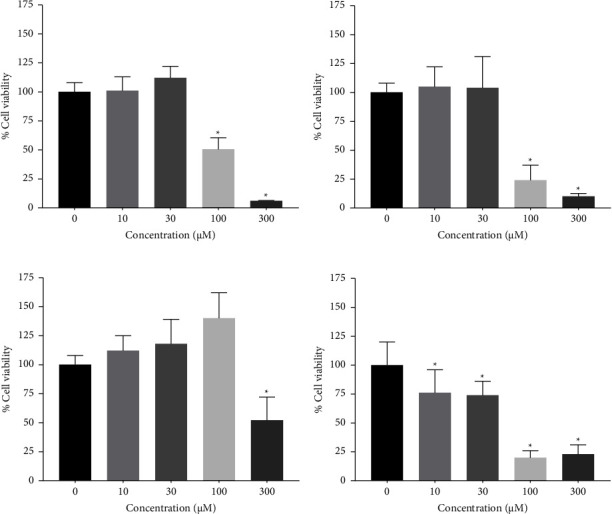
The cytotoxic effect of 0 *µ*M–300 *µ*M oleanolic acid treatment in cell lines A549 (a), HeLa (b), HepG2 (c), and SH-SY5Y (d) after 24 h.

The effect of OA on the cell viability of four different cancer cell lines was evaluated using the MTS assay using concentrations of 0 *µ*M to 300 *µ*M OA. This sapogenin displayed cytotoxic activity in the A549, HeLa, HepG2, and SH-SY5Y cell lines at concentrations equal to or greater than 100 *µ*M (Figure 2). These results indicated that this effect of the mentioned compound increased in a dose-dependent manner. Moreover, the lowest cytotoxic activity was found in HepG2, A549, and HeLa cells, with IC_50_ of 408.3 ± 0.05 *µ*M, 98.9 ± 0.05 *µ*M, and 83.6 ± 0.05 *µ*M, respectively. The highest activity was observed in SH-SY5Y cells, with IC_50_ of 34.1 ± 0.05 *µ*M.

### 3.4. Effect of Hederagenin on Cell Viability in Cancer Cell Lines

**Figure 3:**
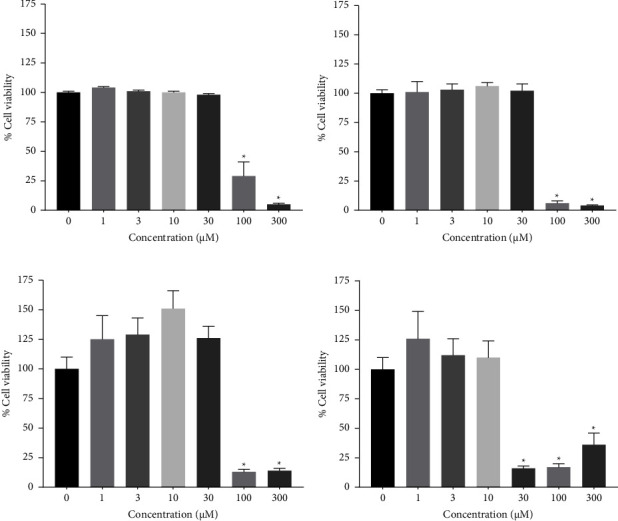
The cytotoxic effect of 0 *µ*M–300 *µ*M hederagenin treatment in cell lines A549 (a), HeLa (b), HepG2 (c), and SH-SY5Y (d) after 24 h.

The cytotoxic effect of hederagenin (HED) at concentrations of 0–100 *μ*M on A549, HeLa, HepG2, and SH-SY5Y cells was evaluated using the MTS assay. We found that the synthetic saponin HED exhibited an efficient cytotoxic effect against A549, HeLa, and HepG2 at 100 *µ*M and SH-SY5Y at 30 *µ*M, indicated by the reduced percentage of cell viability by more than 80% (Figure 3). The IC_50_ values after HED treatment were 78.4 ± 0.05, 56.4 ± 0.05, 40.4 ± 0.05, and 12.3 ± 0.05 *µ*M against A549, HeLa, HepG2, and SH-SY5Y cells, respectively.

### 3.5. Effect of Ursolic Acid, Oleanolic Acid, and Hederagenin, on Caspase-3 Activity Cancer Cell Lines

The results from the treatment of cancer cell lines with UA, OA, and HED sapogenins encouraged us to evaluate whether A549, HeLa, HepG2, and SH-SY5Y cell lines activate the caspase pathway for apoptosis in response to saponin and sapogenin treatment. To verify that the four cell lines do activate the caspase pathway for apoptosis, a preliminary test was performed.

**Figure 4:**
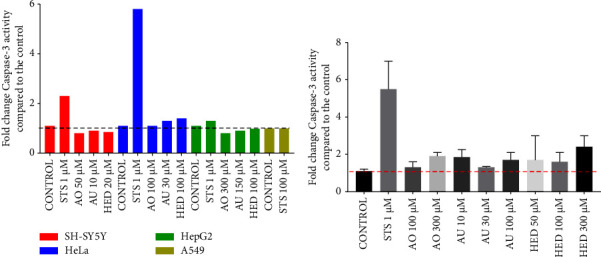
Effect of staurosporine (positive control), oleanolic acid, ursolic acid, and hederagenin on caspase-3 activity in SH-SY5Y, Hela, HepG2, and A549 cell lines (a). Caspase-3 activity in HeLa cells compared with the control (untreated cells) after treatment with different concentrations of sapogenins (b).

Each of the lines was treated with 1 *µ*M STS and found to have higher caspase activity in the positive control (STS) when compared with that in the untreated cells (Figure 4(a)). The only cell line in which no activity was observed even when treated with high concentrations of STS (100 *µ*M) was A549; therefore, it was excluded from subsequent treatments.

On the other hand, the SH-SY5Y, HeLa, and HepG2 cell lines that did present caspase-3 activity were treated at determined concentrations of OA, UA, and HED which were established according to the IC_50_ results obtained from the cell viability assay presented in Table 1. We found that the only cell line that had activity compared to the positive control was HeLa, with concentrations of 100 *µ*M of OA, 30 *µ*M of UA, and 100 *µ*M of HED, respectively, and therefore, SH-SY5Y and HepG2 were not used for further experimentation (Figure 4(a)).

To evaluate the caspase-3 activity in HeLa cells, additional concentrations of the three sapogenins were used as treatments. A one-way ANOVA test and an unpaired Student's *t*-test were used to evaluate the effects of these treatments on caspase-3 activity in these cells. We found that there was a significant increase in caspase-3 production when the cells were treated with the positive control (STS) compared to untreated control cells (Figure 4(b)), thus confirming that this potent, non-selective inhibitor of protein kinases induces apoptosis as a mechanism of cell death [24]. Furthermore, although the treatment with OA induced significant dose-dependent caspase activity (*p* < 0.003), the effect was mild. While only treatment with 100 *µ*M UA showed significant activity when compared to the control, HED had the most significant effect on caspase-3 activity in the HeLa cell line (*p* < 0.02).

### 3.6. Sapogenins Participate in the Activation of Apoptotic Proteins in HeLa Cells

**Figure 5:**
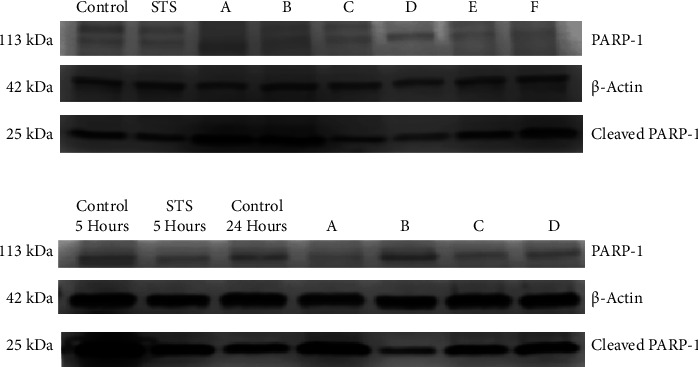
(a) Western blotting analysis of PARP-1 and cleaved PARP-1 expression in HeLa cells treated with A: 100 *µ*M ursolic acid, B: 100 *µ*M oleanolic acid, C: 300 *µ*M oleanolic acid, D: 50 *µ*M hederagenin, E: 100 *µ*M hederagenin, or F: 300 *µ*M hederagenin. (b) Western blotting analysis of PARP-1 and cleaved PARP-1 in HeLa cells treated with A: 100 *µ*M ursolic acid, B: 100 *µ*M oleanolic acid, C: 300 *µ*M oleanolic acid, and D: 50 *µ*M hederagenin for 24 h.

**Figure 6:**
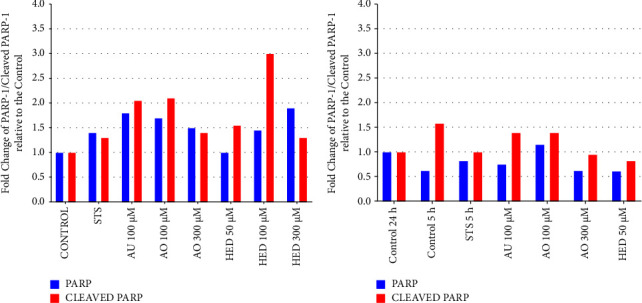
Effect of sapogenins ursolic acid, oleanolic acid, and hederagenin on PARP-1 hydrolysis in HeLa cells for 5 hours, through the western blot method. Western blot method (a). Effect of sapogenins ursolic acid, oleanolic acid, and hederagenin on PARP-1 hydrolysis in HeLa cells for 24 hours by western blot method (b).

Western blotting analysis of HeLa cells treated with UA, OA, and HED for 5 h (Figure 5(a)) showed a series of faint bands for PARP-1 (113 kDa), as well as those for the *β*-actin loading control (42 kDa). Cleaved PARP-1 (25 kDa) bands had greater intensity in samples from cells treated with 100 *µ*M UA and OA when compared with the positive control (cells treated with STS). The other treatments that were evaluated included 300 *µ*M OA, 50 *µ*M, 100 *µ*M, and 300 *µ*M of the HED, apoptosis-inducing positive control (STS), and a negative control (untreated cells). Both UA and OA treatments induced twice as much apoptosis compared to the control at a concentration of 100 *µ*M, indicated by the increased expression of cleaved PARP-1 (Figure 6(a)). These results suggested that caspase-3 cleaved PARP-1 in the late phase of apoptosis. Additionally, when HeLa cells were treated with 100 *µ*M HED, apoptotic activity was three times higher compared to that in the positive control, which is the highest activity observed of the conditions tested and indicated that among all the sapogenins studied, HED had the greatest capacity to induce cell death via apoptosis.

Figure 5(b) presents the results of Western blot analysis of HeLa cells treated with 100 *μ*M UA, 100 *μ*M, and 300 *μ*M OA and 50 *μ*M HED for 24 h. Some of the concentrations after 5 h were excluded because the treatments after 24 h had killed all the cells and the necessary amount of protein could not be obtained for evaluation. Concentrations of 300 *µ*M OA and 50 *µ*M HED did not induce apoptosis after 24 h, indicated by the lower expression of cleaved PARP compared with the control (Figure 6(b)). Conversely, caspase-3 cleaved PARP-1 indicated higher apoptotic activity in cells treated with 100 *µ*M UA and OA than the negative and positive controls.

### 3.7. Thin Layer Chromatography Profile of Saponins and Sapogenins from Red and White Quinoa Extracts

**Figure 7:**
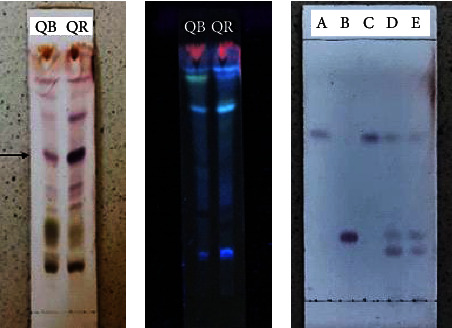
(a) Chromatogram of white and red quinoa to determine the presence of saponins using a Liebermann–Burchard reagent. (b) The presence of a pink-violet stain in the first line and a violet stain in the second one indicates the presence of triterpenoid saponins. In ultraviolet light, at 366 nm, the saponins cannot be observed. (c) Chromatogram comparing the synthetic sapogenins, A: ursolic acid, B: hederagenin, and C: oleanolic acid, to the sapogenin samples from D: red quinoa and E: white quinoa after applying the Liebermann–Burchard reaction.

To demonstrate the presence of saponins in quinoa extracts, we used TLC to separate the biochemical contents of both samples of white and red quinoa extracts.  Figure 7 shows the TLC plates on which various spots indicated that the samples were not purified; however, a pink-purple spot was present in the white quinoa (QB) sample, while an intense violet-colored spot was in the red quinoa (QR) sample. These results are consistent with triterpenoid saponin [25]. The plate was exposed to 366 nm light, which did not show the saponin spots; however, a Retardation Factor (Rf) of 0.46 was obtained for QB and that of 0.58 was obtained for QR.

We also performed TLC to identify the sapogenins after acid hydrolysis using AU, HED, and AO sapogenins (Figure 7(c)). Ursolic acid was observed as a light violet color with a Rf = 0.66 while HED has a violet color with a Rf of 0.38, and OA had a Rf equal to that of UA. The quinoa samples coincided with UA and OA, as well as HED. The Rf values of HED and OA in red quinoa were 0.38 and 0.66, respectively, which were similar to those of white quinoa (Rf = 0.38 for HED and Rf = 0.67 for OA).

### 3.8. Determination of AO and HED in *Chenopodium quinoa* Willd. Extracts

**Figure 8:**
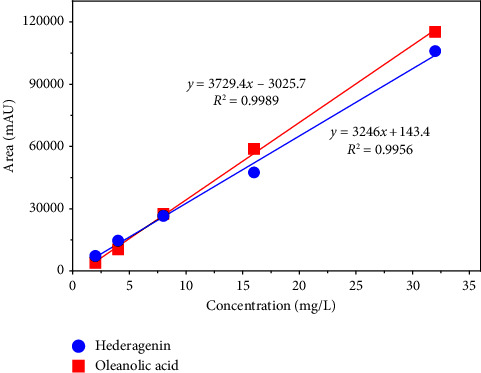
Hederagenin (blue line) and oleanolic acid (red line) calibration curve. The different concentrations were compared against the specific area shown during the HPLC run of each sapogenin.

We conducted HPLC runs in triplicate for each sapogenin and obtained the calibration curve (Figure 8) that enabled us to find the concentrations of sapogenins either AO or HED, for each gram of powder in both kinds of quinoa. OA and HED were 5.61 ± 0.02 mg/L (0.0123 *µ*M) and 9.43 ± 0.02 mg/L (0.020 *µ*M), respectively, in white quinoa. In red quinoa, the HPLC results showed that there were 5.30 ± 0.01 mg/L (0.0116 *µ*M) of OA and 14.44 ± 0.01 mg/L (0.0305 *µ*M) of HED. These results indicated that for every gram of powder residue that is produced when processing white quinoa, there are 2.805 mg of OA and 4.715 mg of HED. Likewise, for each gram of powdery residue that is produced when processing red quinoa, there are 2.65 mg of OA and 7.22 mg of HED.

## 4. Discussion

In this report, we evaluated the cytotoxic activity of ethanolic extracts obtained from white and red quinoa in A549, SH-SY5Y, HepG2, and HeLa cell lines. Our findings demonstrated that both white and red quinoa extracts did not affect the viability of these cells. However, the synthetic sapogenins showed that UA reduced the cell viability percentage at lower concentrations when compared to OA and HED. Furthermore, the IC_50_ values for cells treated with UA were lower compared to OA and HED (Table 1). It has been reported that in HCT15 cells treated with the same concentrations of UA and OA, UA could reduce cell viability more than OA. Similarly, other investigations confirmed the cytotoxic capacity of UA and its derivatives in HL-60, BGC-823, Hela, and Bel-7402 cells [26–28].

While evaluating caspase-3 activity in cell lines treated with saponins, we observed that A549 cells did not activate caspase-3, consistent with previous investigations that reported that this cell line uses autophagy to mediate cell death [29, 30]. Furthermore, we observed that HeLa cells had the highest caspase-3 activity compared to the control (Figure 4(a)). Likewise, analysis in HeLa cells indicated that HED could markedly increase the activity of caspase-3. Previous studies have reported that triterpenoid sapogenins extracted from plants can be cytotoxic in HeLa cells in a caspase-dependent manner [31–33]. Additionally, natural substances isolated from plants have demonstrated cytotoxic activity against cancer cell lines, as demonstrated in a study where they found that the natural flavonoid fisetin induced apoptosis of HeLa cells by triggering the activation of caspases-3 and -8 and the cleavage-PARP, resulting in the induction of apoptosis [34, 35].

When evaluating cleaved PARP-1, HED promoted the highest cleaved PARP-1 activity of the saponins tested. PARP-1 is responsible for DNA damage repair and it is one of the target substrates of caspase-3 [22]. Therefore, the higher the percentage of cleaved PARP-1, the greater the apoptotic activity. Additionally, it was reported that this saponin increased the levels of pro-apoptotic proteins such as cleaved PARP and BAX in head and neck cancer cell lines [36].

By knowing the concentration in mg/L of the sapogenins in both quinoa extract samples, a conversion was made from the concentration units used in the HPLC (mg/L) to the units used in the cytotoxicity assays (*µ*M) to compare the concentrations of sapogenins in extracts versus those that caused a cytotoxic effect in the cell lines studied. The results obtained to explain the lack of cytotoxicity activity of the ethanolic extracts showed that the concentrations of sapogenins were less than 1 *µ*M, while a minimum concentration of 12.30 ± 0.05 *µ*M (Table 1) was necessary to affect viability in these cell lines.

## 5. Conclusions

The ethanolic extracts were obtained from the residues of *Chenopodium quinoa* Willd. and they did not present cytotoxic activity. However, the synthetic sapogenins: UA, OA, and HED, significantly decreased the viability of HeLa, A549, HepG2, and SH-SY5Y cancer cell lines. Similarly, through the caspase-3 assay, it was determined that HeLa undergoes apoptosis during cell death. However, the mechanisms of cell death in lines A549, HepG2, and SH-SY5Y are unknown, and further studies are needed. It was determined that ethanolic extracts of red and white quinoa had higher concentrations of HED than OA. On the other hand, it was found that HED produced a significant increase in PARP-1 hydrolysis in HeLa cells. It would be important to examine in greater depth the function of hederagenin in each of the cell lines, flow cytometry could be implemented to obtain a better cell count during viability, and the use of cellular biomarkers would help to have better information on the biological state of the cell. Taking into account the present research, future studies could focus on studying the composition of the husks of different quinoa varieties since they are wastes of this food that could be a source of sapogenins with potential cytotoxic activity in cancer cells.

## Figures and Tables

**Table 1:** IC_50_ values of cancer cell lines after saponin and sapogenin treatment.

Cancer cell lines	Oleanolic acid (*µ*M)	Ursolic acid (*µ*M)	Hederagenin (*µ*M)
A549	98.9 ± 0.05	21.9 ± 0.05	78.4 ± 0.05
HeLa	83.6 ± 0.05	11.2 ± 0.05	56.4 ± 0.05
HepG2	408.3 ± 0.05	104.2 ± 0.05	40.4 ± 0.05
SH-SY5Y	34.1 ± 0.05	6.9 ± 0.05	12.3 ± 0.05

## Data Availability

All data are included within the article and those not shown are available on request.

## References

[1] Rojas W., Alandia G., Irigoyen J., Blajos J., Santivañez T. (2011). Quinoa: an ancient crop to contribute to world food security. *Bolivia: Oficina Regional de la FAO para América Latina y el Caribe (FAO/RLC)*.

[2] Bhargava A., Shukla S., Ohri D. (2006). Chenopodium quinoa—an Indian perspective. *Industrial Crops and Products*.

[3] Nowak V., Du J., Charrondière U. R. (2016). Assessment of the nutritional composition of quinoa (Chenopodium quinoa Willd.). *Food Chemistry*.

[4] Turcios A. E., Papenbrock J. (2019). Biofiltration of the antibacterial drug sulfamethazine by the species Chenopodium quinoa and its further biodegradation through anaerobic digestion. *Journal of Environmental Sciences*.

[5] Kuljanabhagavad T., Thongphasuk P., Chamulitrat W., Wink M. (2008). Triterpene saponins from Chenopodium quinoa Willd. *Phytochemistry*.

[6] Muir A. D., Paton D., Ballantyne K., Aubin A. A. (2001). Process for recovery and purification of saponins and sapogenins from quinoa (chenopodium quinoa). https://patents.google.com/patent/EP1071441A1/en.

[7] Gawlik-Dziki U., Świeca M., Sułkowski M., Dziki D., Baraniak B., Czyż J. (2013). Antioxidant and anticancer activities of Chenopodium quinoa leaves extracts – in vitro study. *Food and Chemical Toxicology*.

[8] Vilcacundo R., Miralles B., Carrillo W., Hernández-Ledesma B. (2018). In vitro chemopreventive properties of peptides released from quinoa (Chenopodium quinoa Willd.) protein under simulated gastrointestinal digestion. *Food Research International*.

[9] Mohamed D. A., Fouda K. A., Mohamed R. S. (2019). In vitro anticancer activity of quinoa and safflower seeds and their preventive effects on non-alcoholic fatty liver. *Pakistan Journal of Biological Sciences*.

[10] Fan X., Guo H., Teng C. (2023). Supplementation of quinoa peptides alleviates colorectal cancer and restores gut microbiota in AOM/DSS-treated mice. *Food Chemistry*.

[11] Hu Y., Zhang J., Zou L., Fu C., Li P., Zhao G. (2017). Chemical characterization, antioxidant, immune-regulating and anticancer activities of a novel bioactive polysaccharide from Chenopodium quinoa seeds. *International Journal of Biological Macromolecules*.

[12] Shen Y., Zheng L., Peng Y. (2022). Physicochemical, antioxidant and anticancer characteristics of seed oil from three Chenopodium quinoa genotypes. *Molecules*.

[13] Yao L., Lu J., Wang J., Gao W.-Y. (2020). Advances in biosynthesis of triterpenoid saponins in medicinal plants. *Chinese Journal of Natural Medicines*.

[14] Zhao Y., Ma Y., Li J. (2022). Transcriptomics–metabolomics joint analysis: new highlight into the triterpenoid saponin biosynthesis in quinoa (Chenopodium quinoa Willd.). *Frontiers in Plant Science*.

[15] Estrada A., Li B., Laarveld B. (1998). Adjuvant action of Chenopodium quinoa saponins on the induction of antibody responses to intragastric and intranasal administered antigens in mice. *Comparative Immunology, Microbiology and Infectious Diseases*.

[16] Ahumada A., Ortega A., Chito D., Benítez R. (2016). Saponinas de quinua (Chenopodium quinoa Willd.): un subproducto con alto potencial biológico. *Colombian Journal of Chemical-Pharmaceutical Sciences*.

[17] McGowan E. M., Alling N., Jackson E. A. (2011). Evaluation of cell cycle arrest in estrogen responsive MCF-7 breast cancer cells: pitfalls of the MTS assay. *PLoS One*.

[18] Gugnani K. S., Vu N., Rondón-Ortiz A. N., Böhlke M., Maher T. J., Pino-Figueroa A. J. (2018). Neuroprotective activity of macamides on manganese-induced mitochondrial disruption in U-87 MG glioblastoma cells. *Toxicology and Applied Pharmacology*.

[19] Malich G., Markovic B., Winder C. (1997). The sensitivity and specificity of the MTS tetrazolium assay for detecting the in vitro cytotoxicity of 20 chemicals using human cell lines. *Toxicology*.

[20] Pino-Figueroa A., Nguyen D., Maher T. J. (2010). Neuroprotective effects of lepidium meyenii (maca). *Annals of the New York Academy of Sciences*.

[21] Pino-Figueroa A., Vu H., Kelley C. J., Maher T. J. (2011). Mechanism of action of lepidium meyenii (maca): an explanation for its neuroprotective activity. *American Journal of Neuroprotection and Neuroregeneration*.

[22] Jänicke R. U., Ng P., Sprengart M. L., Porter A. G. (1998). Caspase-3 is required for *α*-fodrin cleavage but dispensable for cleavage of other death substrates in apoptosis. *Journal of Biological Chemistry*.

[23] Yue T.-L., Wang C., Romanic A. M. (1998). Staurosporine-induced apoptosis in cardiomyocytes: a potential role of caspase-3. *Journal of Molecular and Cellular Cardiology*.

[24] Zhang C.-L., Wu L.-J., Tashiro S.-I., Onodera S., Ikejima T. (2004). Oridonin induces apoptosis of HeLa cells via altering expression of Bcl-2/Bax and activating caspase-3/ICAD pathway. *Acta Pharmacologica Sinica*.

[25] Hassan H. S., Sule M. I., Musa A. M., Musa K. Y., Abubakar M. S., Hassan A. S. (2012). Anti-inflammatory activity of crude saponin extracts from five Nigerian medicinal plants. *African Journal of Traditional, Complementary and Alternative Medicines: AJTCAM*.

[26] Mazumder K., Tanaka K., Fukase K. (2013). Cytotoxic activity of ursolic acid derivatives obtained by isolation and oxidative derivatization. *Molecules*.

[27] Ma C.-M., Cai S.-Q., Cui J.-R. (2005). The cytotoxic activity of ursolic acid derivatives. *European Journal of Medicinal Chemistry*.

[28] Akihisa T., Kamo S., Uchiyama T. (2006). Cytotoxic activity of Perilla frutescens var. japonica leaf extract is due to high concentrations of oleanolic and ursolic acids. *Journal of Natural Medicines*.

[29] Zhang J., Chiu J., Zhang H. (2013). Autophagic cell death induced by resveratrol depends on the Ca2+/AMPK/mTOR pathway in A549 cells. *Biochemical Pharmacology*.

[30] Hodges A. L., Kempen C. G., McCaig W. D., Parker C. A., Mantis N. J., LaRocca T. J. (2019). TNF family cytokines induce distinct cell death modalities in the A549 human lung epithelial cell line when administered in combination with ricin toxin. *Toxins*.

[31] Wu Z., Wu L.-J., Li L.-H., Tashiro S.-I., Onodera S., Ikejima T. (2004). Shikonin regulates HeLa cell death via caspase-3 activation and blockage of DNA synthesis. *Journal of Asian Natural Products Research*.

[32] Stefanowicz-Hajduk J., Bartoszewski R., Bartoszewska S. (2015). Pennogenyl saponins from Paris quadrifolia L. Induce extrinsic and intrinsic pathway of apoptosis in human cervical cancer HeLa cells. *PLoS One*.

[33] Han L.-T., Li J., Huang F., Yu S.-G., Fang N.-B. (2009). Triterpenoid saponins from Anemone flaccida induce apoptosis activity in HeLa cells. *Journal of Asian Natural Products Research*.

[34] Ying T.-H., Yang S.-F., Tsai S.-J. (2012). Fisetin induces apoptosis in human cervical cancer HeLa cells through ERK1/2-mediated activation of caspase-8-/caspase-3-dependent pathway. *Archives of Toxicology*.

[35] Calandria C., Irurzun A., Barco Á., Carrasco L. (2004). Individual expression of poliovirus 2Apro and 3Cpro induces activation of caspase-3 and PARP cleavage in HeLa cells. *Virus Research*.

[36] Kim E. H., Baek S., Shin D., Lee J., Roh J.-L. (2017). Hederagenin induces apoptosis in cisplatin-resistant head and neck cancer cells by inhibiting the nrf2-ARE antioxidant pathway. *Oxidative Medicine and Cellular Longevity*.

